# Feasibility and integration of an intensive emergency pediatric care curriculum in Armenia

**DOI:** 10.1186/s12245-020-00320-x

**Published:** 2021-01-06

**Authors:** Aline Baghdassarian, Al M. Best, Anushavan Virabyan, Claire Alexanian, Shant Shekherdimian, Sally A. Santen, Hambartzum Simonyan

**Affiliations:** 1 Department of Emergency Medicine Richmond, Virginia Commonwealth University School of Medicine, 1250 East Marshall Street, Main Hospital, 2nd Floor, Suite-600, POBOX: 980401, Richmond, VA 23298 USA; 2 Department of Emergency Medicine Inova Fairfax Campus, 3300 Gallows Road, Falls Church, VA 22042 USA; 3 Department of Emergency and Disaster Medicine, Yerevan State Medical University, Yerevan, Armenia; 4 Georgetown University School of Medicine (M’19), Washington, DC USA; 5 Department of Surgery, University of California Los Angeles, Los Angeles, CA USA; 6 Fund for Armenian Relief 2, Khorenatsi Street, Yerevan, Armenia

**Keywords:** Pediatric emergency, Armenia, EMS, Ambulance, Education

## Abstract

**Background:**

Emergency pediatric care curriculum (EPCC) was developed to address the need for pediatric rapid assessment and resuscitation skills among out-of-hospital emergency providers in Armenia. This study was designed to evaluate the effectiveness of EPCC in increasing physicians’ knowledge when instruction transitioned to local instructors. We hypothesize that (1) EPCC will have a positive impact on post-test knowledge, (2) this effect will be maintained when local trainers teach the course, and (3) curriculum will satisfy participants.

**Methods:**

This is a quasi-experimental, pre-test/post-test study over a 4-year period from October 2014‑November 2017. Train-the-trainer model was used. Primary outcomes are immediate knowledge acquisition each year and comparison of knowledge acquisition between two cohorts based on North American vs local instructors. Descriptive statistics was used to summarize results. Pre-post change and differences across years were analyzed using repeated measures mixed models.

**Results:**

Test scores improved from pretest mean of 51% (95% CI 49.6 to 53.0%) to post-test mean of 78% (95% CI 77.0 to 79.6%, *p* < 0.001). Average increase from pre- to post-test each year was 27% (95% CI 25.3 to 28.7%). Improvement was sustained when local instructors taught the course (*p* = 0.74). There was no difference in improvement when experience in critical care, EMS, and other specialties were compared (*p* = 0.23). Participants reported satisfaction and wanted the course repeated. In 2017, EPCC was integrated within the Emergency Medicine residency program in Armenia.

**Discussion:**

This program was effective at impacting immediate knowledge as well as participant satisfaction and intentions to change practice. This knowledge acquisition and reported satisfaction remained constant even when the instruction was transitioned to the local instructors after 2 years. Through a partnership between the USA and Armenia, we provided OH-EPs in Armenia with an intensive educational experience to attain knowledge and skills necessary to manage acutely ill or injured children in the out-of-hospital setting.

**Conclusions:**

EPCC resulted in significant improvement in knowledge and was well received by participants. This is a viable and sustainable model to train providers who have otherwise not had formal education in this field.

## Background

Acutely ill or injured children pose a considerable challenge to providers worldwide [1–5]. Armenia (29743 km^2^), with a total population of 3 million is a lower-mid income country (LMIC) that gained independence from the Union of Soviet Socialist Republics in 1991. A quarter of the population are children. Yerevan is the capital city, where 30% of the population lives [6]. The emergency medical services system (EMS), in Armenia follows the Franco-German model whereby ambulances are staffed by physician-nurse dyads. This model results in a lower proportion of patients transported to the hospital [1, 7, 8]. In 1995, a 24-month general emergency medicine (EM) residency program was developed to improve emergency and trauma care in Yerevan [9]. Since then, few additional updates have been made to the residency curriculum and out-of-hospital emergency physician (OH-EP) education in Armenia. Moreover, in the more remote provinces of Armenia, the ambulances are staffed by frontline physicians who do not necessarily have any EM training and may be general pediatricians, family practitioners, or surgeons. A needs assessment study conducted in 2012 in Yerevan, identified gaps in pediatric-specific rapid assessment and resuscitation knowledge and skills among the OH-EPs [1].

This study was designed to evaluate the effectiveness of an intensive emergency pediatric care curriculum (EPCC) in increasing the OH-EP’s knowledge in pediatric rapid assessment and resuscitation while applying the train-the-trainer model. Specifically, the aims of the study were first, to show that EPCC, increased immediate post-test knowledge, and achieved positive acceptance by participants. Second, when the curriculum used a train-the-trainer model with local instructors taking over the course, the goal was to determine if knowledge gained was similar in train the trainer and not model.

## Methods

### Study design

This study was conducted over a 4-year period from October 2014 through November 2017. This was a quasi-experimental, pre-test/post-test study of an intensive emergency pediatric care curriculum (EPCC-study intervention) following the train-the-trainer model. During the 4-year period, EPCC was repeated 4 times with different groups of learners. Pre- and post-knowledge tests were conducted each year (2014, 2015, 2016, 2017). The period of 2014 and 2015 (initial cohort, taught by North American instructors/train the trainer) was compared to the intervention period of 2016 and 2017 (instruction by the local instructors). The study was approved by the Ministry of Health (MOH) of Armenia as well as Yerevan State Medical University, Department of Emergency Medical Services. The study was determined exempt by the IRB at Virginia Commonwealth University.

### Study setting

During the study period, in the Yerevan EMS system, there were 185 OH-EPs working with 175 nurses and 125 drivers. In Gyumri, the second-largest city of Armenia (150,000 population), there were 19 OH-EPs. In the rest of the country including Vanatzor, the region of Lori (2017 course), the EMS system is integrated within the polyclinic or hospital systems, and staffed by the on-call physicians in the hospitals or clinics who respond to EMS calls, these physicians do not necessarily have EM training and may be general pediatricians, family practitioners, or surgeons (Dr. A. Virabyan, Personal communication, June 4, 2019).

### Participants

Participants were physicians selected from the respective emergency districts as well as pediatric critical care transport team and the polyclinics (frontline physician providers of rural regions), by our local partners. All physician leaders of their respective teams (EMS district or polyclinic, where the polyclinic constituted the EMS district) were selected to participate. Moreover, when available, all members of the critical care transport team (pediatric critical care providers) were selected to participate.

In 2014, participants were the physician leaders from each EMS district as well as the physicians of the pediatric critical care transport team. All 9 districts of Armenia and the Nagorno Karabakh (Artsakh) Republic were represented.

In 2015, the course was repeated in Yerevan where participants were the OH-EP’s of Yerevan EMS.

In 2016, the course was repeated in Gyumri, Shirak region, and in 2017 in Vanatzor, Lori Region. Here, frontline physicians from the polyclinics, staffing the EMS system were selected to participate.

### Instructors

**Fig. 1:**
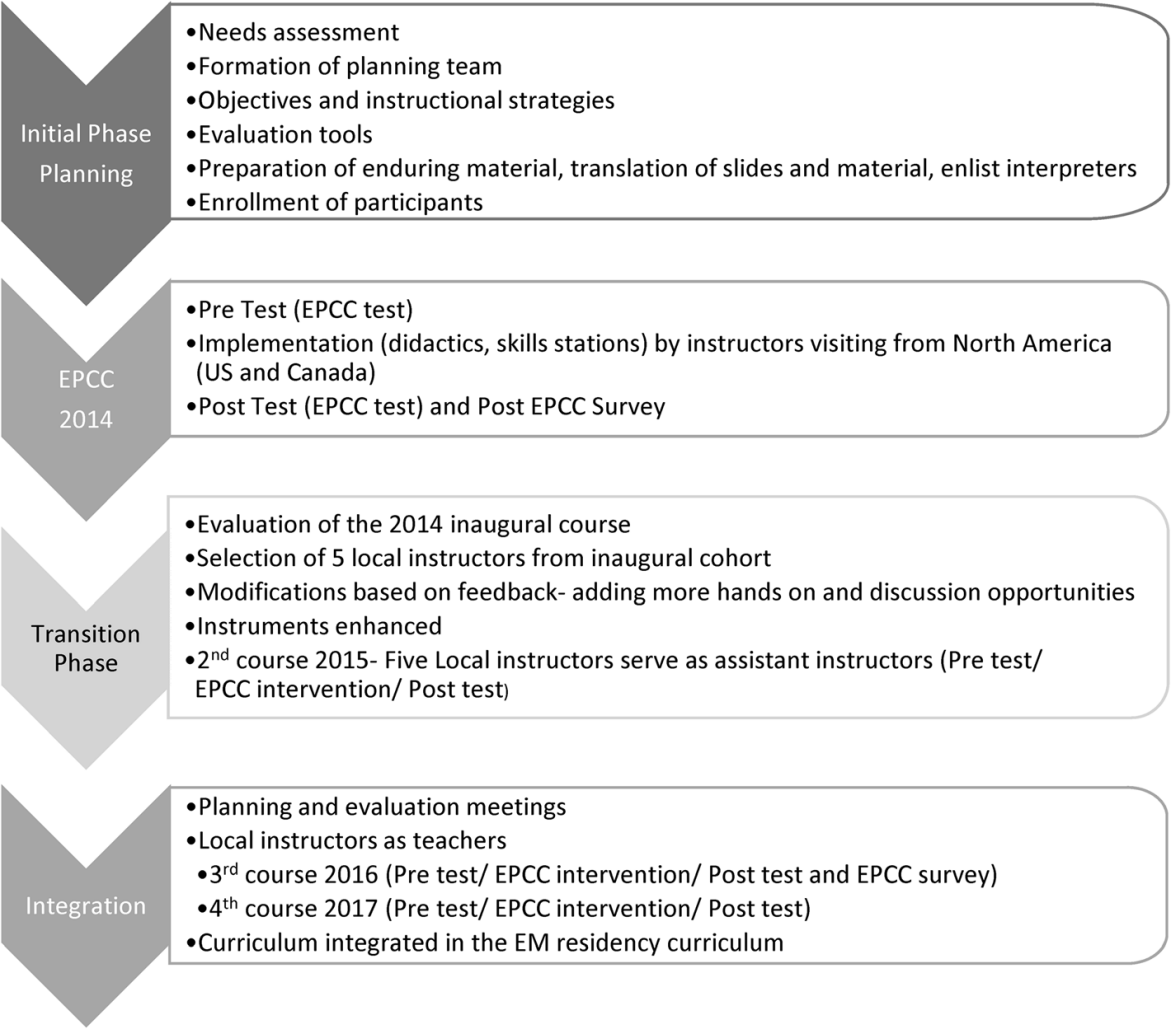
Study flow

From the inaugural cohort of 2014, five local physicians were selected to serve as trainers. These were physicians of the EM residency leadership and the pediatric critical care transport team who successfully completed the course in 2014 and were willing to serve as future instructors. These local instructors served as assistant instructors during the 2015 session to prepare for independent teaching in 2016 and 2017 (Fig. 1).

In 2014 and 2015, EPCC was taught by visiting instructors from North America (United States (US) and Canada) and in 2016 and 2017 by the local instructors in the presence of visiting faculty from North America.

### Curriculum

For curriculum development and course design, we followed the six-step approach described by Thomas, Kern et al. [3] and utilized adult learning theories and principles [10]. The goals and objectives of the course resulted from the targeted needs assessment, as previously published [1]. To help develop course content and implementation strategies, we assembled a team that included leaders, educators, and practitioners from within Armenia as well as North America. Our team included the following:

- From Armenia: leaders of the EM residency program, representatives from the MOH, experts in educational program planning.
- From North America: subject matter experts in Pediatric Emergency Medicine, Pediatric Critical Care, Pediatric Surgery, Pediatric Emergency Nursing.

This process led to the creation of a 37 direct-contact hour curriculum to be conducted over a 5-day period (Table 1). The curriculum followed guidelines for Pediatric Advanced life Support (2010), Advanced Trauma Life Support (2010), Advanced Burn Life Support (2011), crisis Resource Management principles in addition to common themes in pediatric acute and emergency care, identified in the prior needs assessment (Table 1) [5–7]. The course was piloted with five pediatric emergency medicine faculty in the USA and five physicians in Armenia. Feedback was received and modifications were made accordingly.

**Table 1:** Emergency pediatric care course agenda

	Morning	Afternoon
Day 1	Introduction Pretest Understanding children/pediatric assessment Pediatric shock Pediatric sepsis Questions and answers	Respiratory and Cardiac Emergencies Workshop *CPR/defibrillator* (6 stations-concurrent/2 of each) • 1 and 2-rescuer child CPR • 1 and 2-rescuer infant CPR • Defibrillator use Questions and answers Resuscitation team concepts Case discussions • Hypovolemic shock • Distributive shock • Obstructive shock Questions and answers
Day 2	Approach to a trauma patient: Primary survey Secondary survey Case : Pre-hospital trauma Pediatric burns/smoke inhalation Burn case	Pediatric chest trauma Chest trauma case Pediatric abdominal trauma Pediatric head and neck injuries Case: head injury Hands on (group 1) • Vascular access • Pediatric airway management (BVM, laryngoscope, LMA, ETT, oral and nasal airway, O2 delivery devices, Magill forceps) Questions and answers
Day 3	Introduction/recap Neonatal resuscitation Surgical emergencies in neonates Acute abdomen Questions and answers American Heart Association guidelines and updates in cardiopulmonary resuscitation Anaphylaxis	Submersion injuries Coffee break Rashes Ophthalmologic and ear nose throat Questions and answers Hands on repeated (group 2) • Vascular access • Pediatric airway management (BVM, laryngoscope, LMA, ETT, oral and nasal airway, O2 delivery devices, Magill forceps) Questions and answers
Day 4	Introduction/recap Toxicologic emergencies Heat and cold injuries Pediatric metabolic and endocrine Emergencies Emergency medical services for children in the world	Pediatric seizures Questions and answers Workshop • Neonatal resuscitation • Case study (myocarditis, severe croup, anaphylaxis) • Case study (meningococcemia, DKA, heat exhaustion) • IV access • Airway (2 stations) Questions and answers
Day 5	Post-test, course evaluation Review and questions Post test Course evaluation Adjourn	

Over the 5-day period participants were away from clinical responsibilities and dedicated their time to EPCC. The days were divided into morning didactic sessions and afternoon hands on workshops (Table 1). All material (slides, tests, protocols) was translated to Armenian by professional local translators and back translated to English to verify authenticity. All lectures were presented in English and interpreted to Armenian by live interpreters. All lecture material, translated to Armenian, was given to participants on USB drives.

### Assessment of outcomes and evaluation of program

#### Knowledge testing

During each of the 4 years, prior to the implementation of the curriculum, the EPCC test, described below was administered to the participants. The same test was also administered to the participants immediately at the end of the course each year. The 45-item test (EPCC test) consisting of multiple-choice questions was developed by the course faculty based on the learning objectives of the curriculum. Each faculty member preparing educational content to meet the course objectives was asked to provide 4‑5 multiple choice questions pertaining to the topic prepared. Questions were reviewed by an expert in education for content and structure and piloted on a small group of providers both in the US and Armenia for content and clarity (5 from the US and 5 from Armenia). Feedback was received and accordingly integrated. Two questions were dropped due to poor performance. The knowledge questions can be requested from the corresponding author.

#### Course evaluation

Participants were asked to complete a post-intervention survey of their opinions regarding EPCC, whereby they were also asked to include in free text, what changes they would make to their practice as a result of this course. The post-course evaluation forms were adapted from the continuing education office at the Virginia Commonwealth University. Figure 1 represents the study flow.

### Pretest, post-test, and evaluation surveys

All tests and course evaluation forms were translated from English to Armenian by professional translators and back translated to English to maintain accuracy. All tests and evaluation surveys were administered in Armenian and were anonymously completed. Random numbers were assigned to each participant to allow for pairing of pretests and post tests and conduct within-person pre- and post-test paired analysis. Test scores were not shared with the MOH or leadership in Armenia.

### Data collection

EPCC tests were collected on paper. Data was collected on participant primary specialty.

### Statistical analysis

All analyses were performed using the SAS software (SAS version 9.4, JMP Pro version 14.0, SAS Institute Inc., Cary NC). Results were summarized using counts and percentages or means and standard errors, as appropriate. Pre-post change and differences across years were analyzed using repeated measures mixed models. To compare the North American instructors to the train-the-trainer model scores and evaluations were compared across years. The percent correct score was analyzed using repeated-measures ANOVA and the individual item correct-incorrect score was analyzed using repeated-measures logistic regression.

## Results

### Demographics

A total 133 physicians participated throughout the 4 years of the course: 45% of the participants identified EMS as their field of specialty, 30% identified as pediatricians, 12% as critical care, 7% as family medicine, and 6% surgeons (Table 2).

**Table 2:** Physician participant specialty distribution by cohort

	Participants by cohort (*n*)		
Physician specialty	2014‑2015^a^	2016‑2017^b^	All
Critical care	12	4	16
EMS	46	10	56
EMS and family medicine	0	2	2
EMS and pediatrician	0	2	2
Family medicine	0	8	8
Pediatrician	16	23	39
Pediatrician and family medicine	0	1	1
Radiologist	0	1	1
Surgeon	0	8	8
All	74	59	133

^a^Instruction by North American instructors (train the trainer)

^b^Instruction by local instructors

### Knowledge acquisition

**Fig. 2:**
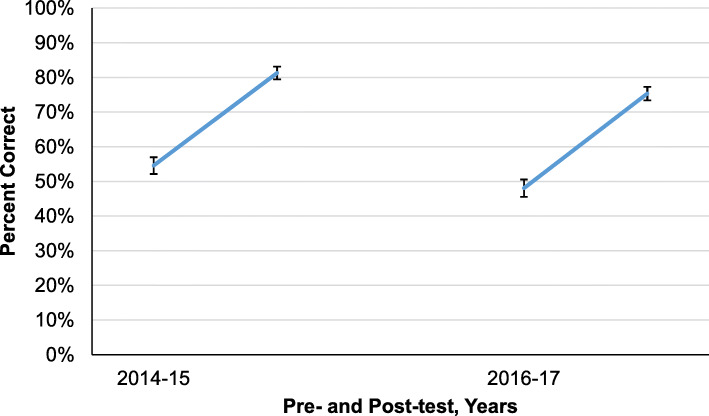
Pretest and post-test percent correct by cohorts

Overall mean pre-test scores of participants were 51.3% correct (95% CI = 49.6 to 53.0%) and post-test scores significantly improved to 78.3% correct (95% CI = 77.0 to 79.6%, *p* < 0.0001). The participants in each year improved by a mean of 27% (95% CI = 25.3 to 28.7%, *p* < .0001), and this did not vary by year (Fig. 2). This improvement was independent of the instructor, either from North America (2014‑2015) or with local physician instruction (2016‑2017) (*p* = 0.74). The amount of improvement also did not vary between specialties (*p* = 0.2293).

**Fig. 3:**
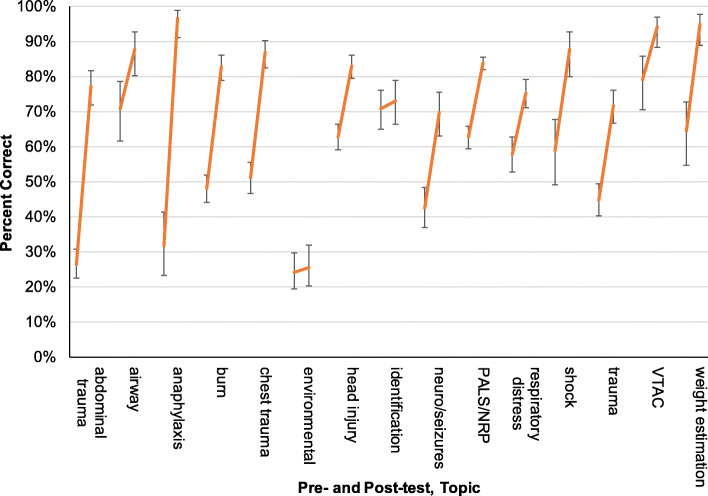
Pretest/post-test % correct by content area

It was also of interest to identify the specific content areas where there was significant improvement. The test items were categorized into 16 content areas covering general pediatric emergencies, resuscitation, trauma, and burns. Significant improvement was identified in the following areas: airway, anaphylaxis, chest trauma, head injury, PALS/NRP, general trauma, abdominal trauma, burn, neuro/seizures, respiratory distress, shock, ventricular tachycardia (VTAC), and weight estimation (Fig. 3). There was insufficient evidence for improvement in the environmental and infectious diseases categories.

### Evaluation survey

Course evaluation surveys, aiming to assess participants’ satisfaction with the course, were collected in 2014 and 2016. In 2014, 91% of participants completed the satisfaction survey, of those 54% had written free-text feedback in the box provided. In 2016, 100% of participants completed the satisfaction survey (course evaluation survey), of those 33% had written free text feedback in the box provided.

Eighty-nine percent were “very satisfied” and the remainder were “satisfied” with the course overall and the format of the conference. All (100%) agreed that participation in this course would improve their patient outcomes, that they would definitely recommend this course to someone else and that they would make changes to their current practice based on participating in this course. Most frequently listed practice changes were systematic approach to trauma patients, use of epinephrine in anaphylaxis and resuscitation, judicious use of antibiotics, and considering evidence when making treatment choices. The free text feedback provided is summarized in Table 3. Overall, participants asked for this course to be repeated, to be brought to their provinces, and to be conducted more frequently. Participants also asked for even more hands-on and discussion opportunities. This last feedback was incorporated in the curriculum.

**Table 3:** Frequency of participant free-text feedback

Summary of participant comments	*n*
Please do these courses more frequently and continue the training	30
Make the duration longer	5
More time for workshops	7
It would be great to organize these for general (clinic) pediatric care	2
Synchronized interpretation would help save time	1

## Discussion

The worldwide scarcity of healthcare providers is compounded by the fact that their skills, competencies, clinical experience, and expectations often do not meet the healthcare needs of the populations they serve [11]. Through a partnership between the US and Armenia, we provided OH-EPs in Armenia with an intensive educational experience to attain knowledge and skills necessary to manage acutely ill or injured children in the out-of-hospital setting.

While our curriculum attempted to span the spectrum of general pediatric emergencies, the course was mainly focused on acute stabilization and resuscitation. The majority of the discussions occurred around acute illness and injuries. Moreover, the knowledge test included one question addressing environmental emergencies (heat-related illness) and one question addressing general infectious diseases (septic joint). This could be a contributing factor to the insufficient evidence of improvement in the categories of environmental emergencies and infectious diseases.

International partnerships have been recognized as imperative to the implementation of emergency medicine (EM) training programs for LMICs [12]. Many examples of short-term educational trips to LMICs have been described in the literature [13–15]. To our knowledge, this is the first program that describes integration within the existing educational system for sustainability. Our program adds a longitudinal view of a curriculum that encompasses general emergency pediatric care and trauma assessment with transition to the local training programs over a 4-year period.

This program was effective at impacting immediate knowledge as well as participant satisfaction and intentions to change practice. This knowledge acquisition and reported satisfaction remained constant even when the instruction was transitioned to the local instructors after 2 years. This program, participant assessment, and program evaluation can serve as a future model for international medical educational programs in emergency pediatric care as well as other specialties with assessed knowledge gaps.

**Fig. 4:**
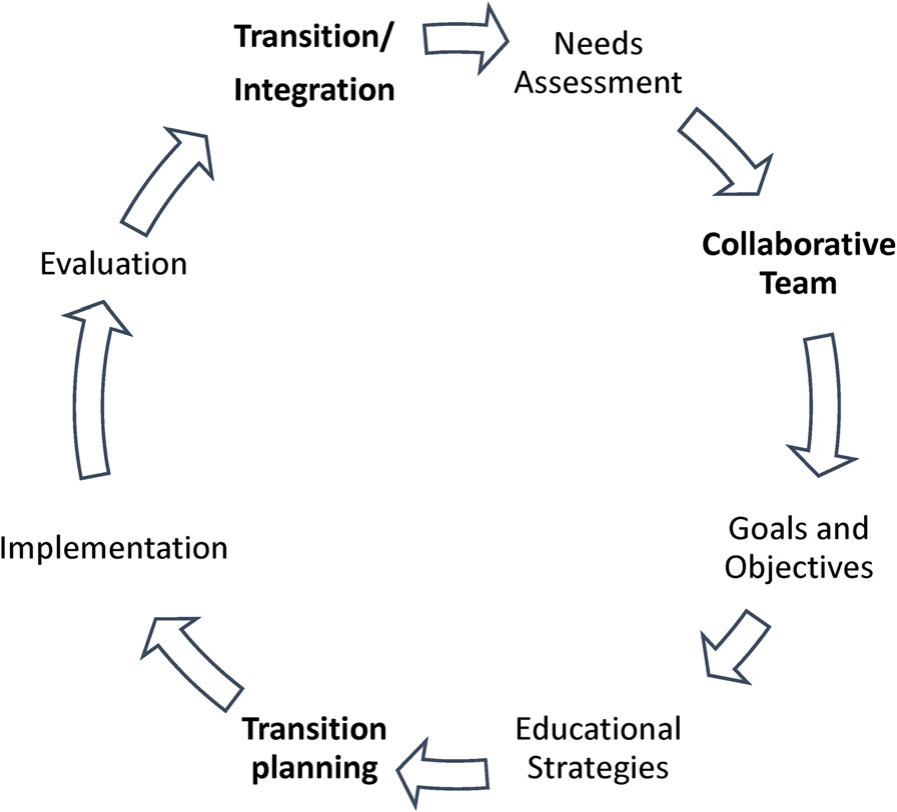
Eight steps to curriculum design in low-mid income country

When designing curricula and educational programs to be implemented in other countries, it is essential to develop a team that includes leaders, educators, and practitioners from within the host country, to address challenges associated with balancing cultural differences, such as language and social constructs. The thorough needs assessment that took place in conjunction with local guidance during the planning and implementation phases, was an essential element for the success of our program. We followed the 6-step framework for curriculum design described by Thomas, Kern et al., and adapted it to our special setting where the initial program pilot was external to the existing educational infrastructure of the country. Figure 4 illustrates our adaptation.

## Limitations and future directions

When comparing the North American instructors to local train-the-trainer model, there was a hybrid year (2015) where the local physicians assisted the international physicians. We chose to include this year in the North American Instructor model which might limit comparisons between groups. Another limitation of this study is the use of immediate knowledge acquisition and learner satisfaction as the metric for the outcomes. In fact, measurement of physician behavior changes and improved patient outcomes would serve as better outcome measures [16]. As the EMS registry database gets organized in Armenia, we intend to measure physician behavior as documented on EMS records. The small sample size of each cohort is also a limitation of this study which will make the results less universally generalizable.

While the course evaluations were anonymous, it is possible that participants had a perceived fear of giving negative feedback. Also, positive feedback may have been influenced by the time off from clinical duties participants were given to attend the course. Evaluations were not collected at the end of 2015 and 2017.

## Conclusion

The intensive and focused educational curriculum in emergency pediatric care, using the train-the-trainer model, was well received by physicians in Armenia, it resulted in significant improvement in knowledge, as well as a perceived positive impact on the practice of participants. This program, successfully integrated within the EM residency curriculum of the country, may serve as a future model for international medical educational programs. There need to be additional studies to evaluate the sustained impact of this intervention further.

## Data Availability

The datasets generated during the current study are not publicly available but are available from the corresponding author on reasonable request.

## Abbreviations

EPCC: Emergency pediatric care curriculum
OH-EP: Out-of-hospital emergency providers
EMS: Emergency medical services
EM: Emergency medicine
LMIC: Low- and mid-income countries
MoH: Ministry of Health
PALS/NRP: Pediatric advances life support/neonatal resuscitation program
VTAC: Ventricular tachycardia
US: United States
